# Development of the WHO-INTEGRATE evidence-to-decision framework: an overview of systematic reviews of decision criteria for health decision-making

**DOI:** 10.1186/s12962-020-0203-6

**Published:** 2020-02-11

**Authors:** J. M. Stratil, R. Baltussen, I. Scheel, A. Nacken, E. A. Rehfuess

**Affiliations:** 1 Institute for Medical Information Processing, Biometry and Epidemiology, Pettenkofer School of Public Health, LMU Munich, Marchioninistr. 15, 81377 Munich, Germany; 2 Department for Health Evidence, Radboud University Medical Center, P.O.Box 9101, 6500 HB Nijmegen, The Netherlands; 3 Department of Global Health, Norwegian Institute of Public Health, PO Box 4404, Nydalen, 0403 Oslo, Norway

**Keywords:** Decision-making, Decisionmaking, Resource allocation, Priority-setting, HTA, Health technology assessment, Criteria, WHO, WHO-INTEGRATE

## Abstract

**Background:**

Decision-making in public health and health policy is complex and requires careful deliberation of many and sometimes conflicting normative and technical criteria. Several approaches and tools, such as multi-criteria decision analysis, health technology assessments and evidence-to-decision (EtD) frameworks, have been proposed to guide decision-makers in selecting the criteria most relevant and appropriate for a transparent decision-making process. This study forms part of the development of the WHO-INTEGRATE EtD framework, a framework rooted in global health norms and values as reflected in key documents of the World Health Organization and the United Nations system. The objective of this study was to provide a comprehensive overview of criteria used in or proposed for real-world decision-making processes, including guideline development, health technology assessment, resource allocation and others.

**Methods:**

We conducted an overview of systematic reviews through a combination of systematic literature searches and extensive reference searches. Systematic reviews reporting criteria used for real-world health decision-making by governmental or non-governmental organization on a supranational, national, or programme level were included and their quality assessed through a bespoke critical appraisal tool. The criteria reported in the reviews were extracted, de-duplicated and sorted into first-level (i.e. *criteria*), second-level (i.e. *sub*-*criteria*) and third-level (i.e. *decision aspects*) categories. First-level categories were developed a priori using a normative approach; second- and third-level categories were developed inductively.

**Results:**

We included 36 systematic reviews providing criteria, of which one met all and another eleven met at least five of the items of our critical appraisal tool. The criteria were subsumed into 8 *criteria,* 45 *sub*-*criteria* and 200 *decision aspects*. The first-level of the category system comprised the following seven substantive criteria: “Health-related balance of benefits and harms”; “Human and individual rights”; “Acceptability considerations”; “Societal considerations”; “Considerations of equity, equality and fairness”; “Cost and financial considerations”; and “Feasibility and health system considerations”. In addition, we identified an eight criterion “Evidence”.

**Conclusion:**

This overview of systematic reviews provides a comprehensive overview of criteria used or suggested for real-world health decision-making. It also discusses key challenges in the selection of the most appropriate criteria and in seeking to implement a fair decision-making process.

## Background

Decision-making in public health and health policy is complex [1–3]. Processes that consider evidence and other considerations in a structured manner require a careful deliberation of many and often conflicting normative and technical criteria [4–10]. The choice of which of these criteria should be employed in the form of criteria has a profound impact on the outcome of the decision-making process. In many decision-making processes directly addressing health, criteria have mostly been concerned with effectiveness and cost [10–13]. This is at odds with the complexity of real-world decision making, were normative and feasibility considerations may act as key drivers of decisions (e.g. infringement of population health interventions on individual rights or interactions of interventions with other components of a health system) [14, 15]. The values and perceptions of different stakeholders with respect to normative and technical considerations often vary greatly both within and across societies. As there are various reasonable and defendable perceptions of which values and principles should guide the decision-making process and as there is no consensus on the *right* or *best* criteria, reasonable disagreement about the *right* decision or action is likely in pluralist societies [16].

Of course, many health-relevant decisions in public health policy and practice are made without adhering to structured decision-making processes based on pre-defined sets of criteria, populating those with evidence and weighting the results. But in various areas of public health policy and practice, such structured processes are relied on in the evaluation or comparison of alternative interventions or modes of actions. This inter alia includes the allocation of resources [9], the setting of research priorities [8, 17], decision-making about public health interventions [18], the assessment of health technologies for funding or reimbursement [19–21], or investment or disinvestment considerations [22]. Selecting the most appropriate and relevant criteria is a challenging but critical task in all of these structured decision-making processes.

The criteria used across different types of decisions have been addressed in multiple reviews. Several reviews have explored the criteria used when applying multi-criteria decision analysis (MCDA) [13, 23–28], an”umbrella term to describe a collection of formal approaches which seek to take explicit account of multiple criteria in helping individuals or groups explore decisions that matter” [29]. Other reviews have explored the criteria employed in the context of health technology assessments (HTA), which intend to examine social, economic, organizational and ethical considerations in relation to health technologies in a comprehensive manner [30]; these covered both the criteria to inform decisions about health technologies by national or sub-national HTA institutions [19, 31–33], and the criteria used for selecting the technologies or interventions a HTA is to be conducted on [8, 34]. In general, reviews have addressed criteria used for making decisions on funding or implementing health interventions or technologies [9, 10, 22, 35–38], prioritizing research topics [39–41] or coverage decisions [7, 12, 42, 43]. Reviews include criteria used on various levels of decision making (national, regional, or local), in different contexts (e.g. high- vs. low-income countries), and proposed by various stakeholder groups (e.g. decision makers, beneficiaries/patients).

Against this background, we conducted this study as part of a larger research project to develop a new evidence-to-decision (EtD) framework. The WHO-INTEGRATE EtD framework was developed to be firmly rooted in WHO norms and values and reflective of the changing global health landscape, and to encompass a comprehensive set of criteria suitable for decision making on clinical practice, public health, and health system interventions [15]. Within the development process of the framework, we conducted this overview of systematic reviews de-novo with the objective to provide a comprehensive overview of criteria used or intended to be used in real-world health decisions. More details on the role of this review in the development process of the WHO-INTEGRATE EtD framework is provided in our publication Rehfuess/Stratil et al. [15].

## Methods

Our search strategy combined the terms “decision-making”, “decision maker*”, “decision analysis”, “multi-criteria decision analysis”, “priority setting”, “resource allocation”, “policy-making” and “policy-maker*” and their synonyms with the terms “criterion” and “criteria” as well as the terms “review*”, “literature search”, “mapping”, “meta analysis” and their synonyms. Searches were conducted in PubMed and focused on the occurrence of these search terms in title and abstract. As the term “criteria” is used in many adjacent fields (e.g. referring to treatment or diagnostic decisions), we complemented these systematic searches with hand searches of the references of all included studies.

Title and abstract screening was conducted using the software Rayyan [44]. Title and abstract screening as well as full-text screening was independently undertaken by two authors (JMS and AN), based on the inclusion and exclusion criteria shown in Table 1. We included studies which had conducted systematic searches of the literature and had comprehensively reported real-world criteria used in health decision-making. Studies focused on clinical decision-making (i.e. concerned with the decisions of individual patients) as well as studies focussing on the concepts or measurements of individual criteria (e.g. cost-effectiveness) were excluded. Where discrepancies could not be resolved by the two screening authors, a third author (ER) was consulted. Screening of the records identified through the updated literature searches was conducted by the author (JMS) and a research assistant (ST). The original literature searches were undertaken in September 2016 and updated in July 2018.

**Table 1:** Inclusion and exclusion criteria for the overview of systematic reviews

Inclusion criteria	Exclusion criteria
The publication is a review based on systematic searches of the literature The publication is concerned with the criteria considered as part of a health decision between two or more options or the weighting of multiple options, made by a governmental or non-governmental organization on a supranational, national, or programme level The health decision is made from a population perspective regarding the general population, patients, healthcare personnel, health decision-makers or other similar stakeholders in public health and healthcare The publication reports on a comprehensive set of criteria identified through the literature searches (at least 3 criteria) The publication is written in English, German, Spanish or Italian	The publication is not a literature review The publication did not utilize a systematic search strategy The publication is focused on selected criteria (e.g. cost-effectiveness) rather than sets of criteria guiding a decision The publication relates to a health decision made outside of an organizational context (e.g. General Practitioner’s office) and/or from an individual perspective (e.g. treatment choices for an individual patient in clinical practice) The publication primarily addresses issues of how to measure, weight or calculate a criterion (e.g. cost-effectiveness, quality of life)

Information extracted from the included reviews were (i) study objective, (ii) type of health decision, (iii) the types of studies included, (iv) the strategy used to identify primary studies or documents, (v) information on how the criteria were compiled in the primary studies, (vi) the topic of the health decision in the primary studies (e.g. public health interventions, pharmaceuticals), (vii) the regional context of the primary studies (e.g. high- or low-income countries), (viii) the decision-making level (e.g. national, regional, local) and, importantly, (ix) the criteria themselves.

We critically appraised included studies. As no adequate, validated critical appraisal tool was available, we adapted items of the CASP systematic review checklist [45] and AMSTAR 2 [46] to our research question (Additional file 1). Our critical appraisal tool focuses on (i) the formulation of a clear research question regarding the decision-making process to be explored, (ii) a comprehensive search strategy, (iii) the adequate selection of eligible studies, (iv) the comprehensive extraction of criteria, (v) the critical appraisal of primary studies, (vi) the adequate description of the identified criteria (vi) the consideration of potential conflicts of interest, and (vii) the use of pre-established methods. The critical appraisal was conducted by one author (JS) and cross-checked by a research assistant (ST).

Given the intended primary use of the WHO-INTEGRATE framework in the development of WHO guidelines, the analysis focuses on substantive criteria (i.e. “What are the considerations or criteria a decision should be based on?”; e.g. cost, health benefit, available resources) rather than procedural criteria (i.e. “How should the process through which a decision is made be organized?”; e.g. transparency, participation of key stakeholders, opportunity for revising decisions).

This information was extracted onto an Excel spreadsheet by one author (JMS) and spot checked by a research assistant (ST). Wherever possible, criteria were extracted as stated in the primary studies. Where the reviews only reported synthesised criteria without a direct link to the primary studies, we extracted these synthesised criteria (e.g. “disease burden” and “burden of illness” as reported in primary studies summarized in a single “burden of disease” criterion in the included review). Categories, referring to the classification system developed or used in the reviews, were not extracted (e.g. “cost” and “cost-effectiveness” as reported in primary studies summarized under the criterion “financial considerations” in the included review). The criteria were then re-organised by one author (JMS) by combining (i) identical criteria (e.g. “burden of disease” and “burden of disease”) and (ii) criteria described through similar terms with the same meaning (e.g. “burden of disease”, “burden of illness” or “disease burden”).

The criteria were then synthesised in a mixed inductive and deductive approach:

For the deductive approach, we used an intermediate step in the development of the WHO-INTEGRATE framework [15], the seven so-called preliminary criteria “Health-related balance of benefits and harms”, “Human and individual rights”, “Acceptability considerations”, “Societal considerations”, “Considerations of equity, equality and fairness”, “Cost and financial considerations”, “Feasibility and health system considerations”, as well as “Evidence considerations” (Note that these categories were revised in the final WHO-INTEGRATE Framework [15]). “Evidence considerations” was singled out to align with the role of evidence as a meta-criterion in the WHO-INTEGRATE Framework: rather than taking evidence as one of several substantive decision-making criteria into account, the framework argues for reflecting on the quality of evidence of each criterion and considering these aspects alongside. We used these—what we refer to as—*criteria* as level one of the category system. During the synthesis, we remained open-minded about revisions of the category system to be able to capture new considerations relevant for decision making in an appropriate manner. For the inductive approach, we started from the criteria as reported in primary studies and reviews and grouped similar criteria into groups of—what we refer to as—*sub*-*criteria* (level two of the category system) and *decision aspects* (level three of the category system). Criteria relating to decision-making principles, procedural criteria and research priority setting were extracted and categorized separately.

In cases where the exact meaning of a criterion was unclear, the primary publication was consulted wherever possible. Were uncertainty remained, these cases were discussed with a research assistant (ST) or with other members of the research team (ER; RB). After an initial sorting of criteria identified through the included reviews into the three-level category system, this was discussed and refined through discussions between JMS, ST, RB and ER; one author (JMS) subsequently conducted a second round of sorting of the extracted criteria to ensure that all *criteria, sub*-*criteria* and *decision aspects* would be placed correctly within the category system.

## Results

The literature search yielded 4448 unique records, of which 106 were assessed for eligibility based on their full text. A further 88 records were identified through hand searching (see Additional file 2 for PRISMA diagram).

We included 36 reviews in this overview of systematic reviews [4–8, 10–13, 19–23, 27, 28, 31–37, 39, 41–43, 47–54]. All of these were published after 2006, with 15 reviews published in 2018 or 2017 and only 5 reviews published before 2010. 16 reviews provided criteria used for or intended to guide various priority setting exercises [5, 6, 8, 11, 12, 31, 32, 34–36, 38, 41, 42, 49, 51, 55], with one review focused on research priority setting (in the field of child health and nutrition) [39]. Six reviews were framed in the context of multi-criteria decision analysis [6, 13, 23, 27, 28, 48]. Three reviews explored criteria used to guide investment or disinvestment decisions [22, 27, 37]. Two reviews assessed criteria to guide the selection of topics for HTA [8, 34], and eight reviews captured criteria used in HTA [8, 11, 19, 20, 31, 32, 48]. Four reviews focused specifically on the evaluation of or decisions on vaccines [4, 21, 47, 55]. 19 reviews exclusively included studies or documents from high-income countries while five had an explicit focus on decision-making processes in low- and middle-income countries [10, 35, 52, 55]. The number of criteria extracted from each publication ranged from 31 [4] to 360 [6].

The *criteria*, *sub*-*criteria* and *decision aspects* based on the -criteria extracted from the reviews are provided in Table 2. An additional category containing synthesized criteria extracted from the included reviews is provided in Additional file 3. The first level of the category system encompasses seven substantive *criteria*, i.e. “Health-related balance of benefits and harms”, “Human and individual rights”, “Acceptability considerations”, “Societal considerations”, “Considerations of equity, equality and fairness”, “Cost and financial considerations”, and “Feasibility and health system considerations”. In addition to these substantive criteria, we also identified an eight criterion “evidence” (Table 3).

**Table 2:** Overview of substantive *criteria, sub*-*criteria,* and *decision aspects*

Criteria	Sub-criteria	Decision aspects
Health related balance of benefits and harms	General considerations surrounding benefit/effect	Benefits/effect/efficacy/effectiveness/impact [1, 2, 4–10, 12–15, 17–27, 29, 33–*36*]
		Health related Benefits/effect/efficacy/effectiveness/impact [1, 2, 4, 8, 11–15, 21, 23, 26, 31, 34, 35]
		Uptake of intervention [15, 20, 34, 35]
		Magnitude of benefit/effect/impact [2, 4, 11, 14, 18, 31, 33, *36*]
		Additional or indirect effects [2, 6, 33, 34]
	Type and composition of effect/benefit/impact [2]	Impact on mortality, survival, longevity and life expectancy [1, 2, 4, 11, 16, 19, 21, 24–26, 28, 34–*36*]
		Last chance therapies [23, 24]
		Impact on morbidity and disability [1, 2, 16, 35]
		Potential changes in health consequences [24, 25]
		Impact on (health-related) quality of life [2, 8, 11, 12, 14, 19, 20, 22, 25, 26, 28, 29, 31, 33, 35, *36*]
		Impact on patient-reported outcomes [2, 12, 16, 21, 26]
		Valuation of health outcomes by patients and desirability of the effects [2]
		Preventive benefits/effects or preventive approaches [1, 2, 4, 5, 16, 21, 25, 26, 31]
	Character of benefit or effect	Onset of effect and time until benefit [2, 11, 13]
		Duration, sustainability and lasting effect [2, 11, 13, 15, 27, 31]
	Individual and population level of benefit	Clinical benefits/effectiveness/impact [2, 3, 5, 11, 14, 17–22, 24, 25, 28, 31–33, *36*]
		Individual level benefit, effectiveness or impact [1, 2, 4, 18, 21, 22, 25, 26, *36*]
		Marginal benefits (for every patient) [20, 26]
		Population level benefit, effectiveness or impact [1–5, 9, 12, 16, 18, 19, 24–27, 31, 34, 35]
		Threshold effectiveness on populations (herd immunity) [15, 34]
	Balance of benefits and harms	Balance of (health) benefits and harms [5, 14, 18, 19, 23, 24, 34]
	General considerations surrounding harm/risk	General safety, risk and tolerability of intervention [2, 4, 5, 7–9, 11–17, 19–26, 29, 31, 33–*36*]
		Magnitude and likelihood of adverse events [26, 33–35]
		Valuation of health outcomes by patients and desirability of the effects regarding harms [34]
		Short and long term risk and safety profile [26]
		Over diagnosis and over treatment [16, 26]
		Stigmatization [2, 26]
		Risk of failure of intervention [15, 34, 35]
		Burden of treatment [2, 26, 33]
		Risk of inappropriate use [8, 16, 25, 26]
		Impact on disease patterns and reduced long-term effectiveness [15, 34]
		Other or additional adverse events [2]
	Health-related need and priority	Health-related needs: in general [1, 2, 11, 14, 22, 23, 24]
		Burden and impact of disease: in general [1, 2, 4, 7–10, 13–16, 21, 23–26, 33, 34, *36*]
		Magnitude of the problem [4, 10, 25, 34]
		Burden of disease measured through epidemiological indicators [1, 2, 4, 5, 8, 11, 13–15, 21, 23–26, 28, 33–*36*]
		Size of affected population and number of potential beneficiaries [1, 2, 4, 5, 8, 10, 11, 13–17, 20–26, 28, 31–34, *36*]
		Maximum potential for disease burden reduction [10]
		Severity of disease/condition: in general [1, 2, 4, 5, 9–12, 14, 16–19, 21–26, 28, 29, 31, 33–*36*]
		Severity of disease/condition: long term outcomes [34, 35]
		Severity of disease/condition: life threatening disease/condition and prognosis without treatment [1, 5, 11, 16, 21, 28, 29, 34]
		Severity of disease/condition: late stage or end-of life status of disease/condition [5, 21, 24, 25, 28, 29, *36*]
		Outbreaks and epidemic potential [15, 34]
		Urgency and emergencies [1, 2, 13, 25]
Human and individual rights		Human rights considerations [2, 11, 12, 20, *36*]
		Autonomy and informed consent [2, 11, 35]
		Privacy and confidentiality [26, 35]
		Intrusiveness of intervention [22]
Acceptability considerations	Perceived priority of the problem	Public perception of disease burden, disease risk or severity [15, 34, 35]
	Acceptability	Acceptability in general [2, 4, 6, 14, 15, 24, 26, 27, 34–*36*]
		Acceptability of cost and financial outcomes [2, 14, 25, 26, 34]
	Acceptability by beneficiaries	Acceptability by beneficiaries: in general [2, 6, 8, 11, 15, 16, 26, 35]
		Comfort, convenience and user experience [2, 11, 12, 14, 15, 20, 24–26, 31, 33, 35]
	Acceptability by those providing intervention	Acceptability by those providing intervention [15, 16, 34, 35]
	Social and cultural acceptability	Social and cultural acceptability [2, 4, 8, 9, 11, 15, 18, 21, 22, 25, 26, 31–34, *36*]
		Ethical/moral acceptability [2, 6, 11, 12, 15, 18, 22, 25, 26, 32–34, *36*]
	Stakeholder demand, interests and pressures	Advocacy and stakeholder (in general) interests and pressures [1, 2, 6, 14, 15, 21, 25, 33, *36*]
		Demands, interest and pressures of the public [1, 2, 8, 14, 24–26, *36*]
		Demands, interest and pressures by industry [2]
		Pressures, demand and interest of beneficiaries and patient representatives [2, 17, 20, 22, 25, 32, 33]
		Pressures, demand and interest of those providing intervention [2, 17, 20, 25, 26, 32, 33]
		Media attention and coverage [1, 32]
Societal considerations	Societal needs and priority	Social burden of disease (individual/population) [8, 18, 21, 29, 34, 35]
		Social needs [24]
		Economic burden of disease on society (in general) [32]
	Social and societal impact	Social impact or benefits [2, 3, 5, 6, 14, 21, 23–26, 31, 33, 35]
		Impact on non-health outcomes (in general) [15, 18, 26, 34]
		Impact on poverty [1, 2, 4, 14, 25, 26, *36*]
		Relevance to social development of the country [10]
		Value of hope [11]
		Raise profile of condition [14]
	Impact on economy	Impact on economy [2, 25, 26]
		Impact on productivity and population in productive age [2, 12, 16, 25]
		Relevance to economic development of the country
		Innovativeness (potential to encourage innovation) [2, 9, 10, 14, 21, 23–25, 31]
	Environmental impact	Environmental and/or ecological impact of intervention (in general) [14, 16, 21, 29–31, 33]
	Impact on future generations	Impact on future generations [19]
Considerations of equity, equality and fairness	Equity and equality	Equity/equality considerations: in general [1–4, 9–11, 14, 15, 18, 19, 22–24, 26, 27, 29, 33–*36*]
		Fairness considerations: in general [2, 3, 6, 11, 16, 26, *36*]
		Impact on (health) (in-)equity/(in-)equality [2, 6, 10, 14, 26, 31, 34, 35]
		Distribution of benefits and harms [10, 26]
	Accessibility	Accessibility in general [2–4, 6, 9, 14–17, 19, 21, 24, 29, 31, 33–35]
		Equity in accessibility [1, 2, 4, 6, 7, 11, 25, 26, 35]
		Physical and spacial accessibility [2, 4, 6, 16, 26]
		Timeliness of access (time spent waiting for treatment) [2, 3, 22, *36*]
		Informational accessibility [11]
		Financial accessibility of intervention/Affordability [4, 9, 10, 12, 14, 19, 25, 26, 34–*36*]
		Affordability: risk of catastrophic health expenditure [11, 16, 25]
		Affordability: cost and Financial impact on beneficiaries [11, 13, 14, 16, 22, 25, 26, 31, 34, 35]
	Availability	Availability/lack of suitable alternatives [1, 2, 5, 11, 12, 14, 17, 19–25, 28, 32, 34–*36*]
		Limitations of alternative interventions [2, 11, 14, 15, 25, 26, 34, 35]
		Rare diseases/orphan disease [12, 14, 16, 21, 24, 28]
		Unmet needs [11, 12, 16, 21, 24, 29]
	Responsibility	Ability to reduce own health risk and conditions arising from patient behavior [4, 5, 11, 16, 24, 25]
	Non-discrimination	Non-discrimination [11, 23, 24]
	Consideration regarding specific populations	Consideration of high-risk populations [2, 11, 14, 26]
		Consideration of vulnerable populations [1, 2, 4, 11, 21, 25, 26]
		Consideration of Socio-economic status [4, 16, 25, 26, *36*]
		Consideration of sex, gender, and/or sexual orientation [2, 6, 16, 25, 26, 34]
		Consideration of race/ethnicity [25, *36*]
		Consideration of care giver responsibilities [29]
		Consideration of age-groups [1, 2, 4, 11, 14, 16, 26, 28]
		Consideration of place of living [11, 16, 25]
		Consideration of identity and ideology [25]
		Other group related considerations [11]
Cost and financial considerations	Financial burden of disease	Financial burden of disease or current intervention on health system [2, 15, 16, 25, 32, 34, 35]
	Cost and budget impact of intervention	Cost/budget impact: in general [1, 2, 4–6, 8, 9, 11–19, 21–23, 25–31, 33–*36*]
		Cost per unit/usage [1, 2, 5, 11, 15, 17, 20, 23–26, 33, 34, *36*]
		Cost over time [20, 21, 25, 32, 34]
		Long term cost/budget impact [25]
		Overall cost/budget impact [1, 2, 11, 22, 26, *36*]
		Direct cost [11, 25, 32, 34, 35]
		Indirect/additional/hidden cost [11, 34–*36*]
		Marginal cost [11, 25]
		Opportunity cost [2, 11, 21, 31]
		Impact on other spending/investments [2, 6, 14, 25]
		Investment/start-up cost [2, 22, 30]
		Operating cost [2, 30]
		Lifecycle cost of intervention/technology [30]
		Economic/financial benefits and cost-minimization potential [1, 3, 8, 11, 13, 22, 25, 27, 28, 34–*36*]
		Cost to/budget impact on government or society [1, 2, 5, 18, 25, 26, 29, 33, *36*]
	Relation of cost and benefits	Relation of cost and benefits [1–7, 9–16, 18–26, 28, 31–*36*]
	Financial context	Appropriateness [2, 14]
	Financial feasibility	Affordability to health system Availability or lack of funds/funding [1–4, 6, 10–12, 14, 15, 21–25, 31, 34, *36*]
	Financial sustainability	Financial sustainability of intervention and consistency of funding [4, 11, 15, 31, 34]
Feasibility and health system considerations	Health-system related needs and priority	Burden of disease on health system [2, 25, 26, 31, 33, 34]
		Needs of the health-system
	Feasibility and capacity to implement	General feasibility considerations [2–4, 7, 9, 10, 14–16, 22–27, 31, 33, 34, *36*]
		Technical feasibility considerations [6, 26, 33]
		Practical feasibility considerations [4, 6, 26]
		Capacity to implement [1, 2, 6, 14, 21, 25, 29]
	Considerations of management and organization of health system	Availability of, capacity of and need for management and organizational structure [1, 2, 16, 22, 26, 34]
		Impact on management and organizational structure [2, 8, 11, 25]
		Logistical considerations [2, 15, 34]
		Availability of, capacity of and need for monitoring, surveillance and information system [34, 35]
	Resource considerations	Availability of, capacity of and need for resources (in general) [1, 2, 10, 14, 30, *36*]
		Impact on resources (in general) [2, 22–24]
		Efficiency of resource use [18, 24, 31]
	Considerations of human resources and their skills	Availability of, capacity of and need for human resources [2, 10, 13, 14, 18, 22, 26, 30]
		Availability of, capacity of and need for skill levels/knowledge of human resources [2, 3, 14, 16, 22, 25, 30, 35]
		Impact on human resources and skill levels [2, 14, 18, 25, 31]
	Considerations of Non-financial physical resources (equipment, infrastructure)	Capacity of, availability of need for of physical resources and infrastructure [16, 18, 22, 25, 26, 34–*36*]
		Impact on non-financial physical resources and infrastructure [18]
	Interaction with and impact on health system	Impact on performance of health system and impact on other services [1, 2, 4, 6, 8, 10, 11, 14, 16, 18, 20, 22, 26, 31, 33–35]
		Interaction and compatibility with other health system components [1–3, 6, 14, 22, 25, 33–35]
		Ease of use, application and burden of intervention [13, 15, 22, 26, 35]
	Appropriateness within health system	Appropriateness (in general) [2, 3, 11, 21, 25, 26, 31, *36*]
		Appropriateness of intervention for specific context [2, 16, 30]
	Legislative and regulatory considerations	Adherence to legal requirements, constrains and implications [2, 8, 10, 15, 16, 18, 25, 26, 31–35]
		Adherence to other directives, standards and requirements [2, 6, 14, 22, 30, 34]
	Political considerations	Political acceptability, interests and pressures [1–4, 6, 10, 11, 14, 18, 21, 25, 26, 33–*36*]
		Donor and global interests and pressures [2, 3]
		Political impact [2, 25, 33, 35]
		Alignment with priorities [1–4, 6, 13–15, 21, 23, 24, 26, 29, 30, 34, 35]
		Mission, mandate and goals of health system [2, 13, 14, 21, 25]
	Strategic considerations	Strategic planning and considerations [1, 2, 13, 19, 25, 31]
		Existing cooperation [2, 10, 14, 22]
		Decisions and practice of other institutions or stakeholders [2, 11, 17, 24, 29, 34]
		Alignment with recommendations, guidelines and standards [1–3, 7, 11, 14, 16, 21, 24, 26, 29, 34]
		Historical context and past decisions [1–3, 11, 13, 14, 17, 19–22, 25]
		Availability of incentives [1, 2]
		Impact on future decisions [6, 14]
		Keeping promises and commitments [2, 13]
	Characteristic of intervention	(technical) Complexity of intervention [2, 4, 6, 16, 21]
		Scalability of intervention [9, 27]
		Ability to evaluate intervention [2, 33–35]
		Reversibility of intervention [2]
		Flexibility of implementation [2]
		Uniqueness [21]
		Frequency of use and expected level of usage/activity [1, 2, 6, 8, 14, 25, 26, 32]
		Dependence on maintenance [26, 30]
		Sustainability of intervention utilization (e.g. Supply of parts, vaccines) [2, 14, 15, 27, 29, 34]
		Position of intervention in care pathway [5, 12, 20, 24]
		Additional uses of intervention [5, 21, 23–25]

1 Youngkong et al. 2009 [10], 2 Guindo et al. 2012 [6]; 3 Waithaka et al. 2018 [36]; 4 Wiseman et al. 2016 [35], 5 Fischer 2012 [43], 6 Wahlster et al. 2015 [13], 7 Ricciardi et al. 2015 [47], 8 Specchia et al. 2015 [34], 9 Hayati 2018 [42], 10 McGregor et al. 2014 [41], 11 MacLeod et al. 2016 [7], 12 Angelis et al. 2018 [48], 13 Barasa et al. 2015 [57], 14 Cromwell et al. 2015 [5], 15 Burchett et al. 2012 [4], 16 Varela-Lema et al. 2016 [38], 17 Vuorenkoski et al. 2008 [12], 18 Cowles et al. 2017 [50], 19 Golan et al. 2011 [33], 20 Erntoft et al. 2011 [51], 21 Friedmann et al. 2018 [28], 22 Ølholm et al. 2015 [37], 23 Stafinski et al. 2011a [19], 24 Stafinski et al. 2011b [20], 25 Mobinizadeh et al. 2016 [32], 26 Marsh et al. 2014 [27], 27 Rudan et al. 2017 [39], 28 Ghijben et al. 2018 [31], 29 Drake et al. 2017 [23], 30 Diaconu et al. 2017 [52], 31 Polisena et al. 2013 [22], 32 Noorani et al. 2007 [8], 33 Johnson et al. 2009 [53], 34 González-Lorenzo et al. 2015 [54], 35 Piso et al. 2009 [21], *36 Niessen et al. 2012* [11]

The publication by Niessen et al. (Ref. 36) is highlighted in italic, as it is the only study meeting all criteria of our critical appraisal tool

**Table 3:** Overview of evidence considerations

Criteria	Decision aspects
Evidence Considerations	evidence in general [1, 2, 11–13, 15, 17, 21, 23, 31, 33, *36*]
	strength of evidence in general [2, 11, 12, 21, 25, *36*]
	Certainty of evidence (in general) [2, 5, 6, 11, 16, 21–24, 26, 28, 33, *36*]
	Quality of evidence [2, 4–6, 11, 14–22, 25, 26, 28, 29, 33, 34, *36*]
	Completeness of evidence [2, 6, 12, 14, 25, 26]
	Validity of evidence [2, 6, 25, 26, 29, *36*]
	Credibility of evidence [29, 34, *36*]
	directness of evidence [2, 11, 28]
	Consistency of evidence [2, 6, 14, 25, 26, 29, 33]
	Precision of evidence effect [2]
	Relevance of evidence [2, 6, 11, 25–29]
	Applicability and generalizability of evidence [2, 11, 22]
	Type and quality of evidence sources [2, 11, 17, 21, 34, 35]
	Experience based evidence [26, 34, 35]
	Evidence requirements [2, 5, 25]

As criteria may be used in different decision-making processes and different decision-making contexts, not all *criteria* may apply. One important distinction, for example, is whether the problem to be addressed (e.g. a specific disease) has already been decided on or not. If so, the decision is about selecting one out of several options to address the problem, and considerations regarding the priority of the problem itself (e.g. burden or severity of disease or disability) are no longer relevant.

As noted in the methods section, we sorted criteria into a category system based on content. This way of organizing the criteria could be modified by adding additional dimensions. For example, one could also adopt a temporal perspective where criteria may relate to the point in time before an intervention is decided on or implemented the process of implementing the intervention or the short-term or longer-term outcome of the intervention. As an illustration, equity considerations can be framed as relating to the starting point (e.g. priority of a given health issue due to high health inequity), as an criterion of relevance to the implementation process (e.g. distribution of adverse events across all those affected by the intervention) or as an outcome (e.g. reduced health inequity several years after introducing the intervention). Further additional dimensions could be a focus on individuals, populations or systems (e.g. clinical health benefits for the individual, reduction of the disease burden of a population, or impact on the performance of a health system following an intervention). In the organization of the criteria, we kept such additional organizational dimensions in mind.

The most frequently reported criteria were health-related impact of interventions, cost, cost-effectiveness and political interests or priorities; these were covered in all of the included reviews. Rarely used criteria were concerned with the environmental or societal impacts of interventions, and (non-financial) resource availability/needs. The granularity (level of detail with respect to *sub*-*criteria/aspects*) varied widely depending on the criterion: the criteria related to cost or financial considerations included general (e.g. “resource use” or “cost”) as well as very specific usages (e.g. distinct ways to quantify cost-effectiveness). In contrast, criteria related to the societal or environmental impacts of interventions, as well as considerations regarding equity or equality were usually reported in very generic terms.

Evidence in general or evidence regarding specific criteria was mentioned in most included reviews, most often using generic terms, such as “evidence”. In some cases, the criterion evidence referred to specific measures, primarily “evidence of effectiveness/efficacy” and sometimes “evidence on cost”. In other reviews, this included criteria regarding the relevance of the available evidence for a given context (e.g. “relevance of evidence” or “generalizability of evidence”) and criteria regarding the quality of evidence (e.g. “certainty of evidence”, “credibility of evidence” or “validity of evidence”) (see Table 3 and Additional file 4).

We also identified several considerations of specific relevance to research priority setting, covering considerations regarding the answerability of the research question, research ethics or avoidance of duplication of research. As those were not the primary focus of this publication, they are not further discussed here but listed in Additional file 4.

Furthermore, the included publications reported several decision-making *principles* (i.e. guiding concepts from which different criteria derive). The distinction between decision-making principles and substantive criteria is not always clear cut. For example, *human rights* can be regarded as an underlying principle from which other criteria derive (as used in the human rights-based framework by Bustreo et al. [56]), as well as a specific criterion (assessing whether the intervention is in accordance with human rights). From the publications included in our overview of systematic reviews, the following criteria were extracted: Beneficence, non-maleficence, fairness, diversity, fair innings, proportional shortfall, concern for the worse off, justice, formal justice, social justice, distributive justice, principles of human rights, principle of human dignity; marginal utility principle, principle of need and solidarity, collectivism, cohesion, mutuality, rule of rescue and Rawls’ difference principle (see Additional file 4).

Table 2 provides an overview of the *criteria, sub*-*criteria* and *decision aspects* for the seven substantive criteria. Criteria relating to evidence are reported in Table 3.

The results of the critical appraisal are provided in Additional file 5. Only one publication, Niessen et al. [11], met all eight items; 11 out of 36 publications met five or more items. Most publications did not conduct a critical appraisal of included studies, did not report independent extraction of criteria by two reviewers, and did not state explicitly, that the review had been undertaken based on a protocol or otherwise pre-established methods (Additional file 5).

## Discussion

### Summary of findings

Drawing on 36 included reviews, we identified a set of 200 unique *decision aspects*. These were sorted into 7 substantive *criteria* und 45 *sub*-*criteria* as well as a separate criterion on evidence. The substantive criteria cover health-related balance of benefits and harms; human and individual rights; acceptability considerations; societal considerations; considerations of equity, equality and fairness; cost and financial considerations; and feasibility and health system considerations. We found that some *criteria, sub*-*criteria* and decision *aspects* are well developed in the literature, such as those referring to the health implications of an interventions or to the costs of an intervention. In contrast, several others lacked a clear conceptualisation, notably those relating to societal implications or equity and equality considerations.

The wide range of *decision aspects* were used to refine the *criteria* and *sub*-*criteria* in the WHO-INTEGRATE EtD framework, as well as to inform the development of definitions and guiding questions provided as part of the framework.

In addition to their use in the WHO-INTEGRATE framework, we postulate that the list of *criteria, sub*-*criteria* and *decision aspects* can be helpful to decision-makers in their own right: To the best of our knowledge, this is the most comprehensive and up-to-date list of real-world criteria available for health decision-making. It could therefore provide a valuable tool for informing decision-makers wishing to select those criteria relevant for a given type of decision and decision-making context. This comprehensive list is likely to be most relevant to decisions in public health or healthcare. Due to the focus of the present study, the applicability for research priority setting or the evaluation of diagnostic or testing devices is likely to be more limited, as we may not have covered all relevant publications.

Most of the reviews included in our overview of systematic reviews did not meet all or even a majority of the items of our critical appraisal tool. This finding does, however, need to be interpreted in view of the following considerations. First, a validated critical appraisal tool appropriate for the topic does not exist—neither at the level of systematic reviews nor at the level of primary studies. Only three of the included reviews undertook some form of critical appraisal: Whaitaka et al. [36] and Burchett et al. [4] used an adapted CASP Qualitative Checklist and Niessen et al. [11] used custom quality-of-research assessment scales. Second, the low score of some of the included reviews is likely due to poor reporting rather than poor conduct (e.g. regarding pre-established methods, or data extraction in duplicate).Third, the value and relevance of criteria for a given decision-making process does not necessarily depend on the quality of the review they were derived from. For example, even if the criterion “environmental impact” was merely mentioned in a single systematic review of low quality, this would not invalidate its relevance for a decision-making process focused on interventions with pronounced environmental (adverse) effect (e.g. large-scale usage of DDT in malaria prevention).

### Contextualization of findings

With our overview of systematic reviews, we build on several previously published reviews, notably, the review by Guindo and colleagues, which represented the most extensive general overview of criteria until now [6]. Rather than focusing on specific decision-making processes (e.g. priority setting in low- and middle-income countries), we sought to cover the full range and heterogeneity of criteria and their use across various health fields.

We followed an approach focusing on descriptive (“what criteria are used?”), rather than prescriptive (“what criteria should be used?”) approach. Several overviews of more prescriptive frameworks have been published in the field of public-health ethics in recent years [58–60]. A similar undertaking—providing decision-makers with a basis to select appropriate criteria—was conducted by Vermeulen and Krabbe, who provided an overview of the most widely recognized arguments and principles used in decision-making [18]. Their more prescriptive publication, which explores decision arguments and principles, and our more descriptive publication complement each other.

In contrast to some of the other reviews of criteria for decision making [6, 10], we abstained from quantifying how often criteria were cited for several reasons: First, the focus of this publication was to provide an overview of criteria that can be used for decision-making, rather than to provide an overview of which criteria are (widely) used in different decision making settings, as was the purpose in other publications [5, 6, 35]. Second, the quantification of how often or rarely a criterion is used does not necessarily imply its relevance for a given decision-making process: we believe that relevance should be informed by normative considerations. Third, there is a pronounced heterogeneity in the included studies: this begs the question, whether a criterion used in decision-making in a local hospital should count as much as the criteria used in the health technology assessment process of a national or supra-national organization. Finally, the quantification of the use of criteria is complicated: not only were many studies cited in several included reviews [61], but some of the reviews referred to other reviews as their data sources [6, 38].

### Strengths and limitations

Our focus of the literature search on a single data base (PubMed) and the reliance on a selection of terms such as “criteria/criterion” might have missed relevant studies conducted on this issue. These decisions were made due to significant time and resource constraints relating to the development of the WHO-INTEGRATE framework over a relatively short period of time. We countered this potential limitation by thoroughly searching the references of all included studies, which yielded some additional publications. Furthermore, during the extraction of the criteria from included reviews we noted that we seemed to have reached saturation, as from the mid-way point, additional extracted studies yielded no or minimal additional criteria. Expanding the search to additional databases, especially those in the fields of political sciences and health economics, with a more inclusive search strategy may yield valuable additional insights from a broader range of disciplines.

A significant strength of our publication is that—to the best of our knowledge—it is the most extensive overview of criteria used in or proposed for health decision making. We included studies from several different health fields, conducted on various levels of decision-making and topics and in heterogeneous contexts around the world. We classified this comprehensive and diverse set of criteria according to a theory-based categorization system comprising three levels, i.e. *criteria, sub*-*criteria* and *decision aspects*. In doing so, as a team we critically reflected on extracted criteria and their underlying rationale, seeking to be as consistent as possible in how we sorted criteria reported in included reviews into higher-order categories.

### Implications for policy and practice

The very large number of criteria and sub-criteria identified in this publication highlights the complexity of health decision-making It can serve as a resource when considering which criteria to include in sound multi-criteria approaches (i.e. adhering to principles of completeness, lack of redundancy, mutual independence, operationalizability and clustering) and how to use these.

#### The challenge of selecting the *right* criteria

At the centre of any decision-making process will be the challenge of who selects which criteria and how they should be weighted or ranked against each other. As various stakeholders with diverging but reasonable motives are likely to disagree on which criteria are the *right* ones, the focus often shifts from selecting the *right* criteria to making decisions using a *good* or *fair* process [16, 62]. Numerous procedural conditions which characterize such a fair process have been proposed, including in the Accountability for Reasonableness framework [16], among others [63–66]. A fair and transparent process and especially an adequate representation and participation of all relevant stakeholder groups is essential for achieving legitimacy [62, 63].

One approach to overcoming reasonable disagreement about criteria for decision making is to reflect on the underlying normative principles and to make them explicit, e.g. by exploring the roots of a conflict which may lie in (potentially) conflicting normative arguments, e.g. if improving the life of a large number of people has to be weighed against the interest of those suffering from rare diseases with no alternative treatment. While we extracted such principles in our overview of reviews, others have focused explicitly on these [18, 58, 60] and several frameworks to guide the discussions and selection process have been proposed in the public health ethics literature [59, 60, 64, 65, 67–74].

Furthermore, underlying motives and drivers of stakeholders should be taken into account when reflecting on proposed criteria, as these can manifest themselves as trojan horses cloaked in ethical rhetoric [75]. Some calls for strengthening the consideration of criteria beyond evidence of effectiveness or incremental cost-effectiveness ratios are motivated by vested interest in a specific outcome [27, 76]. Such conflicts of interest should not necessarily lead to dismiss the arguments made, but it should lead to a critical reflection regarding the relevance and appropriateness of the proposed criteria for a given decision-making process and the power relations in the discourse [75, 77].

#### The challenge of resolving conflicts within and between criteria

The criteria by themselves are often highly interconnected and at times conflicting. An example is the criterion “age”, which can serve as a “surrogate” criterion for other normative and (harder to measure) considerations. For example, a focus on interventions targeting younger people may be motivated by their potential for achieving a longer life span (greater health impact) or to reduce productivity losses (positive impact on the economy). At the same time, “age” can be considered with respect to non-discrimination or equity: explicitly reflecting on age in order not to prioritize one group of people based on age as a characteristic (ageism). An explicit reflection on and discussion of such conflicts within criteria is important.

Furthermore, the criteria identified in the included reviews are partly overlapping (e.g. cost, effectiveness, and cost-effectiveness). Depending on the decision-making process and the tools used (e.g. MCDA), accounting for overlaps and redundancies may be of relevance. This can, for example, be achieved through selecting non-overlapping criteria or through increasing the granularity of the criteria. In particular the MCDA-literature has developed methods and guidance on how to identify and handle overlapping criteria [78].

The example of “age” as a criterion that can have conflicting interpretations highlights the need to set up a mechanism for handling conflicts within criteria and balancing interests in place. The same holds true for conflicts between criteria (e.g. positive impact on population health, negative impact on the natural environment), which occur on a regular basis in decision-making processes.

#### The challenge of using criteria

Populating criteria with evidence presents a third important challenge [15]. Evidence collection and synthesis approaches are well developed for some criteria (e.g. health impacts) although some challenges remain. For a few criteria, approaches are virtually non-existent in the literature on health decision-making (e.g. environmental implications) while for others there is a lack of clarity regarding the best methods to be employed (e.g. societal or environmental impact assessments) [15]. It is highly likely that suitable methods exist outside of the health decision-making or broader healthcare and public health literature and learning from other disciplines may offer solutions to this challenge.

Guideline development, HTA and other decision-making processes aiming to integrate evidence and criteria for decision making in a structured manner usually operate under significant time and resource constraints. To avoid treating criteria beyond effectiveness and cost-effectiveness as an “after thought”, evidence will need to be collected or analysis on these other criteria. This will require the development of rapid and pragmatic approaches to keep such decision-making processes feasible.

## Conclusion

The comprehensive list of criteria from and for real-world health decision-making presented here was an essential building block in the development of the WHO-INTEGRATE framework. We postulate that it can also be a useful stand-alone tool to inform health decision-making processes not employing an EtD framework. To make the best possible use of this list, solutions to the challenges of selecting criteria, of resolving conflicts between criteria or their interpretation, and of identifying and appraising evidence towards these criteria will need to be found. The WHO-INTEGRATE framework seeks to address some of these challenges, by providing a set of criteria selected based on a strong normative basis and by offering a methodological toolbox, which suggests both comprehensive and pragmatic approaches to populating criteria with evidence [15].

## Supplementary information

**Additional file 1.** Critical appraisal tool.

**Additional file 2.** PRISMA flow diagram.

**Additional file 3.** Overview of criteria on abstraction levels 1–3 and levels 1–4.

**Additional file 4.** Overview of evidence considerations and principles.

**Additional file 5.** Results of the critical appraisal of included studies.

## Data Availability

All data generated or analyzed during this study are included in this published article and its Additional files.

## Abbreviations

MCDA: Multi criteria decision analysis
EtD: Evidence to decision
HTA: Health technology assessment
