# Supplementary material for: Development of the WHO-INTEGRATE evidence-to-decision framework: an overview of systematic reviews of decision criteria for health decision-making
Source: Cost Eff Resour Alloc. 2020 Feb 11;18:8. doi: 10.1186/s12962-020-0203-6 (PMC7014604; doi:10.1186/s12962-020-0203-6)
Supplement: Supplementary file 1 — Additional file 1. Critical appraisal tool. [file 12962_2020_203_MOESM1_ESM.pdf]

Additional file 1: Critical appraisal tool

Judgment for the items:

o Yes	o Partially yes	o No	o Unclear

Items of the critical appraisal tool:

**1.0**	Did the review address a **clearly focused question** in regards to the decision-making process to be explored?	
1.1	Was the type of decision-making process clearly defined?<br>(e.g. priority setting, resource allocation, decision-making process employing MCDA)	
1.2	Was the topic of the decision-making process clearly defined?	
1.3	Were the institutions or stakeholders to be supported in the decision-making process clearly defined?	
**2.0**	Did the review employ a **comprehensive search strategy**?<br>Whether a search strategy is adequate depends on the research question. While items 2.1 to 2.5 inform this judgement, they do not all have to be answered positively for a search strategy to be judged as comprehensive.	
2.1	Were at least two relevant databases searched?	
2.2	Was a hand search of the references conducted?	
2.3	Were attempts undertaken to identify grey literature?	
2.4	Were experts consulted?	
2.5	Were key words and/or a search strategy provided?	
**3.0**	Was the **selection of** eligible **studies adequate**?	
3.1	Did the review authors perform all stages of the screening of publications in duplicate?	
3.2	Were inclusion and exclusion criteria provided?	
**4.0**	Did the review critically **appraise the included primary studies** or assess whether included primary studies followed a rigorous process to identify criteria?	
**5.0**	Were approaches undertaken to ensure that **all criteria** were **extracted comprehensively** from the publications included in the review?	
5.1	Did the review authors perform the extraction of the criteria in duplicate?	
5.2	Did the review extract all criteria from the primary studies, rather than only a sample or sub-set of criteria?	
**6.0**	Are the identified **criteria adequately described** in the review?	
6.1	Does the study list criteria extracted from the primary publications?	
6.2	Is it possible to track back all criteria to a primary publication?	
6.3	Does the review describe the included studies in adequate detail to locate the criteria in context?	
**7.0**	Can it be assumed that a **conflict of interest** has **not** influenced the results?	
7.1	Did the review authors report any financial or non-financial sources of potential conflict of interest?	
7.2	Can it be assumed that there is **no** risk that a conflict of interest could have biased the criteria selected and reported in the review?	
**8.0**	Does the review contain an **explicit statement** that the **methods** were **established prior** to the **conduct of** the **review** and did the report justify any significant deviations from the protocol?	
