# Supplementary figures and images for: Development of the WHO-INTEGRATE evidence-to-decision framework: an overview of systematic reviews of decision criteria for health decision-making
Source: Cost Eff Resour Alloc. 2020 Feb 11;18:8. doi: 10.1186/s12962-020-0203-6 (PMC7014604; doi:10.1186/s12962-020-0203-6)

## Additional file 2 - PRISMA Flow Diagram

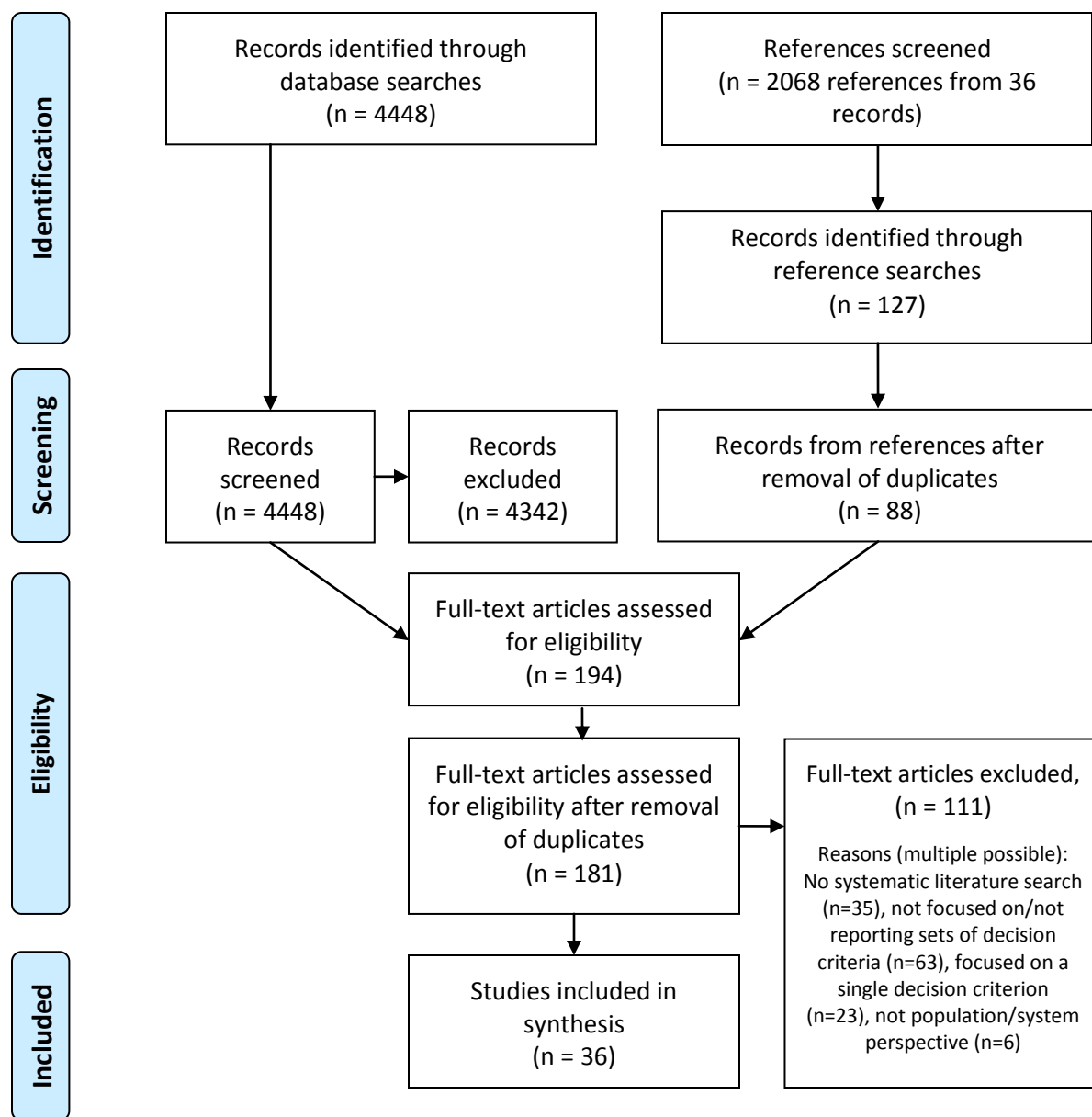

Supplement: Supplementary file 2 — Additional file 2. PRISMA flow diagram. [file 12962_2020_203_MOESM2_ESM.pdf]
