# Supplementary material for: Development of the WHO-INTEGRATE evidence-to-decision framework: an overview of systematic reviews of decision criteria for health decision-making
Source: Cost Eff Resour Alloc. 2020 Feb 11;18:8. doi: 10.1186/s12962-020-0203-6 (PMC7014604; doi:10.1186/s12962-020-0203-6)
Supplement: Supplementary file 5 — Additional file 5. Results of the critical appraisal of included studies. [file 12962_2020_203_MOESM5_ESM.pdf]

Additional file 5 – Results of the critical appraisal of included studies

Study ID	Angelis 2018	Barasa 2015	Burchett 2012	Cowles 2017	Cromwell 2015	Diaconu 2017	Erntoft 2011	Fischer 2012	Friedmann 2017
1.0 Clear research question	Yes	Yes	Yes	Yes	Yes	Yes	Partially yes	Yes	Partially yes
2.0 Comprehensive search strategy	Yes	Yes	Yes	Yes	Yes	Yes	Partially yes	Yes	Yes
3.0 Adequate selection of eligible studies	No	No	No	No	Partially yes	Yes	Partially yes	Partially yes	Yes
4.0 Critical appraisal of eligible studies	No	No	Yes	No	No	No	No	No	No
5.0 Comprehensive extraction of criteria	No	No	Yes	No	No	Partially yes	Partially yes	Partially yes	No
6.0 Adequate description of criteria	Partially yes	Yes	Partially yes	Yes	Yes	Partially yes	Partially yes	Yes	Yes
7.0 Consideration of conflict of interest	Yes	Yes	Yes	Yes	Yes	Yes	Yes	Yes	Yes
8.0 Pre-established methods	Partially yes	No	No	No	Partially yes	Yes	No	No	No

Study ID	Ghijben 2017	Golan 2010	González-Lorenzo 2015	Gunido 2012	Hayati 2018	Johnson 2009	McGregor 2014	MacLeod 2015	Marsh 2014
1.0 Research question	Yes	Partially yes	Yes	Partially yes	Yes	Partially yes	Yes	Yes	Partially yes
2.0 Search strategy	Yes	No	Yes	Yes	Yes	Partially yes	Yes	Yes	Yes
3.0 Selection of eligible documents	Yes	No	Yes	No	Partially yes	No	No	Yes	Yes
4.0 Critical appraisal	No	No	No	No	No	No	No	No	No
5.0 Extraction of criteria	Yes	No	Yes	No	No	No	No	No	No
6.0 Description of criteria	Yes	Partially yes	Yes	Yes	Yes	Yes	Yes	Yes	Yes
7.0 Conflict of interest	Yes	Yes	Yes	Yes	Yes	Yes	Yes	Yes	Yes
8.0 Pre-established methods	No	No	No	No	No	No	No	No	No

Study ID	Mobinizadeh 2016	Niessen 2012	Noorani 2007	Ølholm 2015	Piso 2009	Polisena 2013	Ricciardi 2015	Rudan 2017	Specchia 2015
1.0 Research question	No	Yes	Partially yes	Yes	Yes	Yes	Yes	Partially yes	Yes
2.0 Search strategy	Yes	Yes	Yes	Yes	Yes	Yes	Yes	Yes	Partially yes
3.0 Selection of eligible documents	No	Yes	Yes	Yes	No	Yes	Yes	No	No
4.0 Critical appraisal	No	Yes	No	No	No	No	No	No	No
5.0 Extraction of criteria	No	Yes	No	No	No	Partially yes	Partially yes	No	No
6.0 Description of criteria	Partially yes	Yes	Partially yes	Yes	Yes	Yes	Yes	Yes	Yes
7.0 Conflict of interest	Yes	Yes	Yes	Yes	Yes	Yes	Yes	Yes	Yes
8.0 Pre-established methods	No	Yes	No	No	No	No	No	No	No

Study ID	Stafinski 2011a	Stafinski 2011b	Varela-Lema 2016	Vuorenkoski 2008	Wahlster 2015	Waithaka 2018	Wiseman 2016	Youngkong 2009
1.0 Research question	Yes	Yes	Partially yes	Yes	Yes	Yes	Yes	Yes
2.0 Search strategy	Yes	Yes	Partially yes	Yes	Yes	Yes	Yes	Yes
3.0 Selection of eligible documents	Yes	Yes	Yes	No	Partially yes	No	No	Yes
4.0 Critical appraisal	No	No	No	No	No	Yes	Partially yes	No
5.0 Extraction of criteria	Yes	Yes	No	No	No	No	No	No
6.0 Description of criteria	Yes	Yes	Yes	Yes	Yes	Yes	Yes	Yes
7.0 Conflict of interest	Yes	Yes	Yes	Yes	Yes	Yes	Yes	Yes
8.0 Pre-established methods	No	No	Partially yes	No	Partially yes	No	Partially yes	No
